# Rapid drug detection equipment based on molecular imprinting and surface plasmon resonance technology

**DOI:** 10.3389/fchem.2025.1645843

**Published:** 2025-09-11

**Authors:** Hong Jiang Yu, Wei Li Luo, Xuan Liu, Nan Li, Qing Bo Kong

**Affiliations:** 1 Department of Criminal Science and Technology Chongqing Police College, Chongqing, China; 2 Department of Investigation Inner Mongolia Police College, Hohhot, China; 3 Qingdao ASent Technology Development Co., Ltd., Qingdao, China

**Keywords:** molecular imprinting technique, surface plasmon resonance, methamphetamine hydrochloride, drug detection, sensor chips

## Abstract

This study develops high-sensitivity drug detection equipment combining molecular imprinting technology (MIT) and surface plasmon resonance (SPR) for rapid methamphetamine hydrochloride (MAPA) analysis. The MIT-SPR sensor chip exhibits a detection limit of 2.97×10^−12^ g/mL (0.003 ng/mL), maintains 98.5% sensitivity after 50 days, and achieves 97.3–101.7% recovery in dust samples. The chip demonstrates excellent reusability (8 adsorption-elution cycles) and specificity, with minimal cross-reactivity to structural analogs. Three field-deployable devices were designed for wastewater monitoring, multi-channel sampling, and portable dust detection, enabling real-time data transmission to law enforcement. Compared to traditional methods (e.g., GC, HPLC), this system offers superior sensitivity, faster response (6–10 min), and minimal sample pretreatment. The technology supports diverse sample types (sewage, organic liquids, dust) and reduces false alarms, enhancing anti-drug operations.

## 1 Introduction

By the end of 2023, China had 896,000 registered drug users (Drug Situation Report for 2003, 2023) including 455,000 methamphetamine users who account for approximately 51% of the total number of drug users. The situation of drug abuse remains a serious issue as it continues to damage public health and social stability. Accurate methamphetamine detection and control are critical research priorities (Hongxia and Chen, 2013; Xu, 2010; Yu et al., 2024). At present, the commonly used methods in laboratories for drug detection include gas chromatography (Meng et al., 2004), high-performance liquid chromatography (Duan, 2018; Liu Y. et al., 2018), gas chromatography combined with mass spectrometry (Yao, 2006; Zhang, 2006), and thin layer chromatography (Cheng and Huang, 1997; Xu et al., 2003). However, the above methods have many drawbacks, such as expensive equipment, complicated sample pretreatment process, long detection cycle, and non-real-time detection. In areas with severe drug situations and border regions, the sample submission process is time-consuming and laborious, which may delay the opportunity to act due to the limited number of police personnel (Geng et al., 2015). Currently, the commonly used rapid drug detection technologies include the colloidal gold method, enhanced Raman spectroscopy, and the biological analysis method. The colloidal gold method is easily affected by various external factors, such as taking ephedrine-containing drugs, pH of the analyte, concentration of the sample, and detection time. The above-mentioned reasons may lead to false-positive or false-negative test results (Meng et al., 2016). The enhanced Raman spectroscopy has poor reproducibility due to the randomness in the substrate material preparation. Moreover, the electrode preparation conditions are relatively strict, the operation is complex, the substrate storage is difficult, the preparation flexibility is poor, and the cost is high, which is not suitable for the detection of the current diverse new drugs (Chen et al., 2018; Yang et al., 2016). The biological analysis method has disadvantages such as insufficient specificity, poor stability, and complex sample pretreatment. Therefore, it is particularly important to develop a simple and efficient rapid drug detection device. The molecular imprinting technique (MIT) is a technical method used to simulate the specific identification of biomolecules. It is a polymer with specific selectivity designed according to the spatial structure and recognition sites of the target molecules. This polymer has presetting, selectivity, and recognition for target molecules. Moreover, it can be used for specific detection of a certain drug and excludes interference of other substances with similar structures (Li et al., 2024; Lin et al., 2024). SPR refers to a kind of electron sparse wave which spreads along the surface of metal at a certain depth (usually less than 300 nm). It is a kind of quantum optoelectronics phenomenon that can detect the physical and chemical properties of metal surface substances by observing the changes in the refractive index (Cui et al., 2007; Ouyang et al., 2024). This technology has outstanding advantages, including high sensitivity, a fast detection process, less sample demand, and non-labeling. Using MIT as the recognition element in SPR sensors enhances the sensor’s specificity, anti-interference capability, and reusability. Currently, the MIT–SPR integrated technology is widely applied in detecting proteins, biological toxins, and agricultural/veterinary drug residues, demonstrating advantages such as high sensitivity, simple operation, low energy consumption, and real-time analysis (She, 2010; Yang, 2017; Yang et al., 2007). The convergence of MIT and SPR technologies in this work enables a new class of drug detection platforms with enhanced molecular discrimination capabilities. Engineered field devices demonstrate unprecedented sensitivity (LOD = 2.97 × 10^−12^ g/mL), outperforming conventional chromatography by 66-fold (Zhang et al., 2008). Integrated real-time surveillance and cloud-based data transmission empower law enforcement with rapid response capacity against illicit drug operations.

## 2 Technical introduction

### 2.1 Preparation of molecular imprinting polymers (MIPs)

**FIGURE 1:**
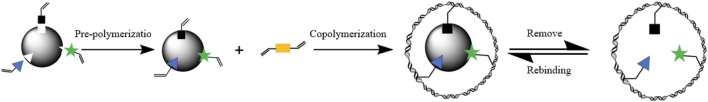
The preparation process of MIPs.

In some solvents, template molecules and monomers form a complex by covalent bonds, non-covalent bonds, or other interactions. Then, cross-linking agents and initiators are added to the solution. The polymerization is initiated using light, heat, electricity, and other methods. Therefore, cross-linking agents, monomers, and other substances bond together to form a rigid polymer with some mechanical strength. Finally, the template molecules in the polymer are eluted using an eluent. A structure that completely matches the template molecules in the spatial position and binding sites is formed. The cavities formed in the polymer are well-matched with the imprinting molecules in space structure, size, intermolecular interaction, and hydrogen bonds. Polymers prepared using this method can specifically recognize the imprinting molecules. The synthesis process of molecular imprinting polymers (MIPs) is shown in Figure 1.

In this study, methamphetamine hydrochloride (MAPA)-imprinted polymers (MAPA-MIPs) were synthesized *in situ* via photo-initiated polymerization using methacrylic acid (MAA) as the monomer, acetonitrile as the porogen, ethylene glycol dimethacrylate (EGDMA) as the cross-linker, and benzophenone (BP) as the initiator. The dynamic processes of film formation, elution, and adsorption were monitored using an SPR sensor chip.

### 2.2 Detection principle

This study uses frequencies specifically selected to avoid resonance with natural light, environmental noise, and electronic components. A lock-in amplifier locks onto, processes, and analyzes the laser signal. Changes on the chip surface are investigated by quantifying the intensity differential between the incident light and the output light following SPR absorption.

## 3 Experiments

### 3.1 Instruments and reagents

Instruments: vacuum coating instrument (Shenyang Huiyu Technology Co., Ltd. Shenyang, China), LaSFN9 prism (Sichuan Juke Optical Technology Co., Ltd. Chengdu, China), ultraviolet light source (UV-LED, λ = 365 nm, 2w/cm2), SPR angle scanning sensor (independently developed), analytical balance (Shanghai Xiniu Laibo Instrument Co., Ltd. Shanghai, China), and constant flow pump (YZ1515).

Reagents: dodecyl mercaptan (Sinopharm Reagent, 98%), methacrylic acid (Sinopharm Group, 98%), ethylene glycol dimethacrylate (McLean reagent, 99%), methamphetamine hydrochloride (Shanghai Yuansi Standard Technology Co., Ltd., chemical standard/reference substance), benzophenone (Chinese medicine reagent, 98%), acetic acid (macrocyclic reagent, 99.5%), anhydrous ethanol (Century Star Chemical Reagent, 99.7%), and phosphoric acid (Hengxing Reagent, 85%).

### 3.2 Preparation of MAPA-MIP sensor chips

The sensor chips included LaSFN9 prism substrate, gold film, and MAPA-MIPs.

1. Prism cleaning: The impurities in the base material had a significant influence on the quality of the gold film and the sensitivity of the SPR sensor chips, so the prisms should be thoroughly cleaned before coating. The prism surface was wiped repeatedly with absorbent cotton until no obvious fingerprints, water stains, and solid impurities remained; then cleaned with distilled water and anhydrous ethanol, respectively; and finally blow-dried with nitrogen. The selection of metals and optimization of film thickness for chip surfaces have been comprehensively documented in our previous work and thus will not be revisited in this study (Tan, 2015).
2. Au film deposition: A gold film of 50 nm was evaporated on the bottom of the prism through vacuum evaporation, and it was soaked in ethanol solution containing dodecyl mercaptan (1 mmol/L) for 24 h to ensure complete self-assembly of dodecyl mercaptan on the gold film surface. Then, it was rinsed with anhydrous ethanol and dried using nitrogen.
3. MAPA-MIP synthesis: The sensor chips with MAPA-MIPs were prepared through *in situ* photo-grafting. The preparation process was as follows:
  - At 25 °C, 14.9 mg of MAPA as the template molecule and 34.45 μL of MAA as the functional monomer were dissolved in 4 mL of acetonitrile. Then, the reactants were evenly mixed using ultrasonic shock for 10 min. The reactants were placed for 3 h to ensure the complete self-assembly process of monomers and template molecules.
  - A volume of 192.5 μL crosslinking agent EGDMA and 8.0 mg initiator BP were added and fully mixed after ultrasonic shock for 10 min. The reaction tank and pipeline were cleaned using pure acetonitrile first, and then, the reactants were injected into the reaction tank using a constant flow pump controlling the flow rate. The flow rate was kept as slow as possible to prevent the gold film from being washed out.
  - The SPR turntable was adjusted to a preset angle, and the back of the reaction tank was illuminated using a UV light source. The changes in reflectivity during the synthesis process were observed, and the UV lamp was turned off when the reflectivity decreased to the lowest point (approximately 90 min). Detailed procedures for the template-to-monomer ratio optimization are provided in Supplementary Material Section S1.
4. NIP synthesis: The non-molecular imprinted polymers (NIPs) without template molecules were synthesized using the same method. The specific adsorption of MAPA-MIPs was compared with that of NIPs.

## 4 Results and discussion

### 4.1 Characterization of self-assembled films using SPR

Air and acetonitrile were used as the medium to characterize the change in the resonance angle before and after self-assembly. First, the bare gold chip was added into the SPR sensor, and the resonance angle was scanned in air and acetonitrile. Similarly, the change in the resonance angle after self-assembly with dodecyl mercaptan was detected (the experimental results are shown in Table 1). The dielectric constants of the media were different. The inflection points and resonance angles of the gold film in the two media were also different. The resonance angle of the gold film in air and acetonitrile increased after self-assembly, indicating formation of the self-assembled film.

**TABLE 1:** The resonance angle of the gold film before and after self-assembly in acetonitrile and air.

Medium	Inflection point	Resonance angle before self-assembly	Resonance angle after self-assembly	Changed value
Air	22.35	28.88	29.12	0.24
Acetonitrile	46.03	58.51	58.80	0.29

**FIGURE 2:**
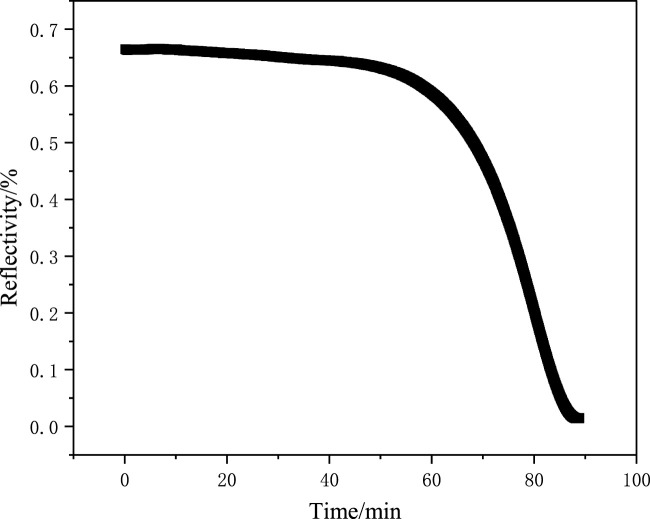
Kinetic curve of the SPR sensor chip.

**FIGURE 3:**
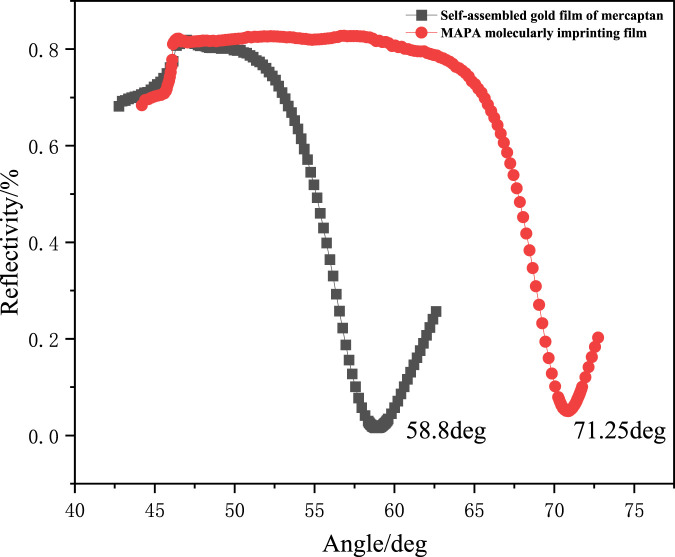
Angle scanning diagram of the SPR sensor chip before and after polymerization.

The formation process of MAPA-MIPs was observed using an SPR sensor. The polymerization kinetics curve and SPR angle scanning curve before and after film formation are shown in Figures 2, 3.

According to the previous research results of our research group (Liu, , 2009; Qingquan, 2011; Tan, 2015; Tan et al., 2020), when the preset resonance angle was 71°∼72°, the phenomenon of surface plasmon was more sensitive. In this experiment, 71.4° at 60% reflectivity was selected as the target resonance angle to monitor the formation process. The kinetic curve of the reaction is shown in Figure 2. It can be observed from Figure 2 that the reflectivity decreased to the minimum when the UV light source was irradiated for 87 min. When the SPR of the incident light and MAPA-MIPs was most significant, the UV lamp was turned off. After the reaction, pure acetonitrile was injected into the pipeline. The angle scanning test was performed on the sensor chip, and the test results are shown in Figure 3. The deviation of 0.15° from the preset resonance angle of the sensor chip was due to the different refractive indices of acetonitrile and the prepolymer solution.

### 4.2 Elution performance

**FIGURE 4:**
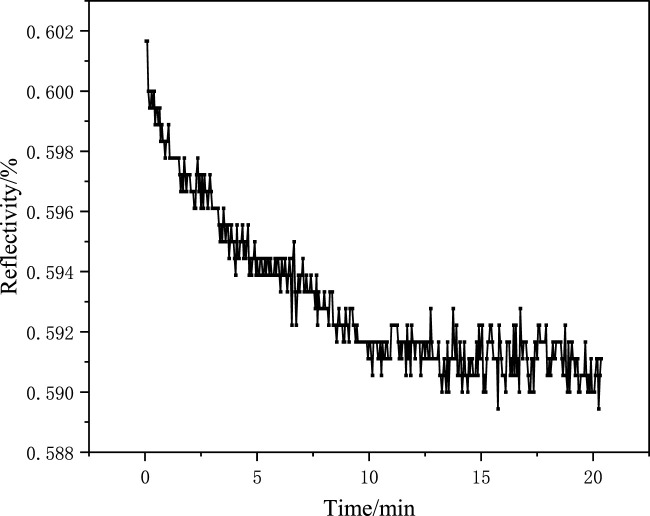
Elution kinetic curve of the SPR sensor chip.

**FIGURE 5:**
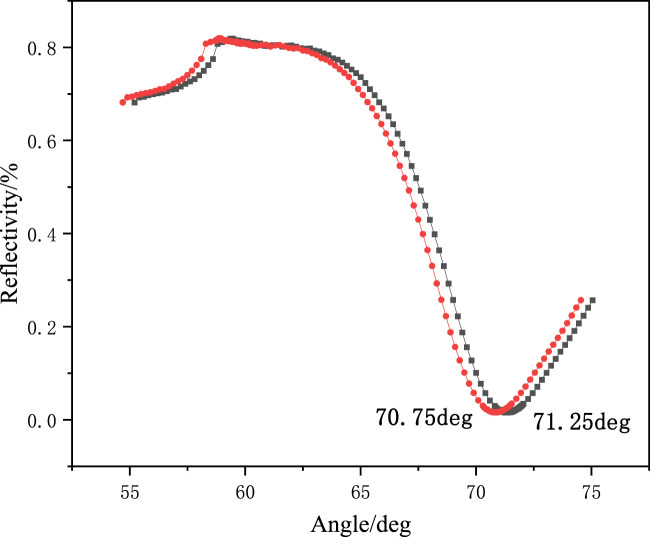
Angle scan of the SPR sensor chip before and after elution.

Acetic acid eluent (acetic acid/water = 1: 9, v/v) was prepared, and the MIF with template molecules was eluted. The elution performance of MIF was monitored using an SPR detector at 60% reflectivity. The elution kinetic curve is shown in Figure 4. After the eluent was added to the reaction tank, the reflectivity began to decrease because the hydrogen bonds formed between the template molecules and monomers were destroyed by the eluent. The template molecules gradually fell off from the MIF. Many holes were formed on the MIF, and thus, the reflectivity was reduced. It could be observed that the resonance angle of the sensor chip changed significantly from 71.25° before elution to 70.75° after elution, as shown in Figure 5. The resonance angle was decreased by 0.5°. It was because the refractive index of the MIF with holes was reduced.

### 4.3 Adsorption performance

According to the previous research results of our research group, the MIF with MAA as the monomer underwent the most sensitive change in reflectivity when the pH of the solution was 5 (Tan, 2015). Therefore, the pH of distilled water used in this adsorption experiment was adjusted to 5 using phosphoric acid.

**FIGURE 6:**
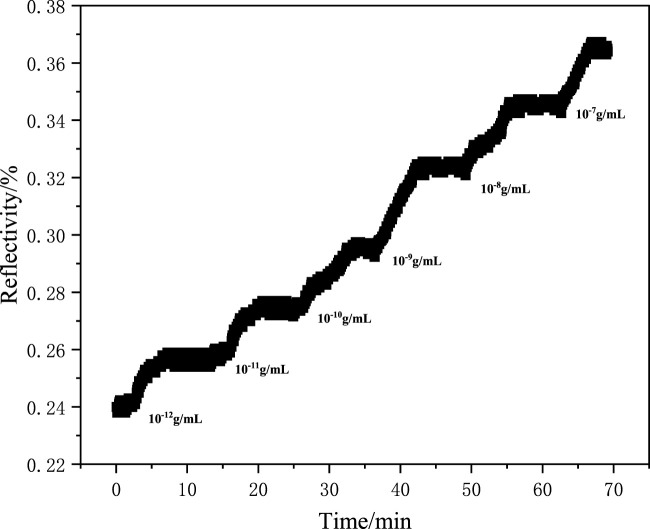
Adsorption kinetics curve of the SPR sensor chip.

**FIGURE 7:**
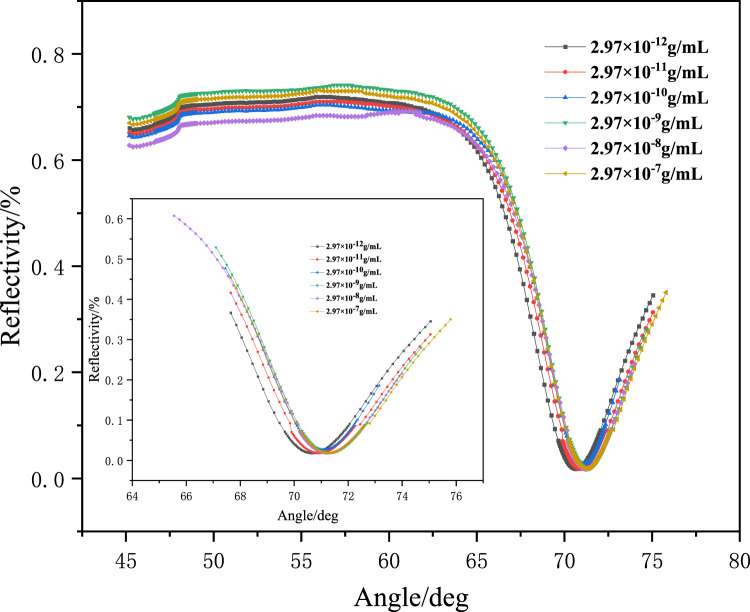
Angle scanning diagram of the SPR sensor chip before and after adsorption.

A series of aqueous solutions of methamphetamine hydrochloride with different concentrations were prepared, and the solution concentrations were 2.97 × 10^−12^ g/ml, 2.97 × 10^−11^ g/ml, 2.97 × 10^−10^ g/mL, 2.97 × 10^-9^ g/ml, 2.97 × 10^−8^ g/mL, and 2.97 × 10^−7^ g/mL. The adsorption experiment of the MIF was carried out at 20% reflectivity, and the change in reflectivity was monitored using an SPR detector. The result is shown in Figure 6. As shown in Figure 6, the reflected light intensity exhibits concentration-dependent enhancement, confirming the high adsorption capacity of the molecularly imprinted polymers for target analytes. The resonance angle of the SPR sensor chip was scanned after the adsorption experiment. The change in the resonance angle after adsorption of different concentrations of solutions was observed, as shown in Figure 7. As shown in Figure 7, a concentration-dependent rightward shift in the resonance angle was observed as the adsorption solution concentration increased. This result indicates that the MIP membranes prepared using the ultraviolet grafting method have extremely high detection limits and detection ranges. The detection limit can reach 10^-12^ g/mL, and the monitoring range is 10^−12^–10^−7^ g/mL. In addition, the experiments on detecting drug residues in human saliva and dust and the quantitative analysis experiment of the SPR sensor chip are still ongoing.

**FIGURE 8:**
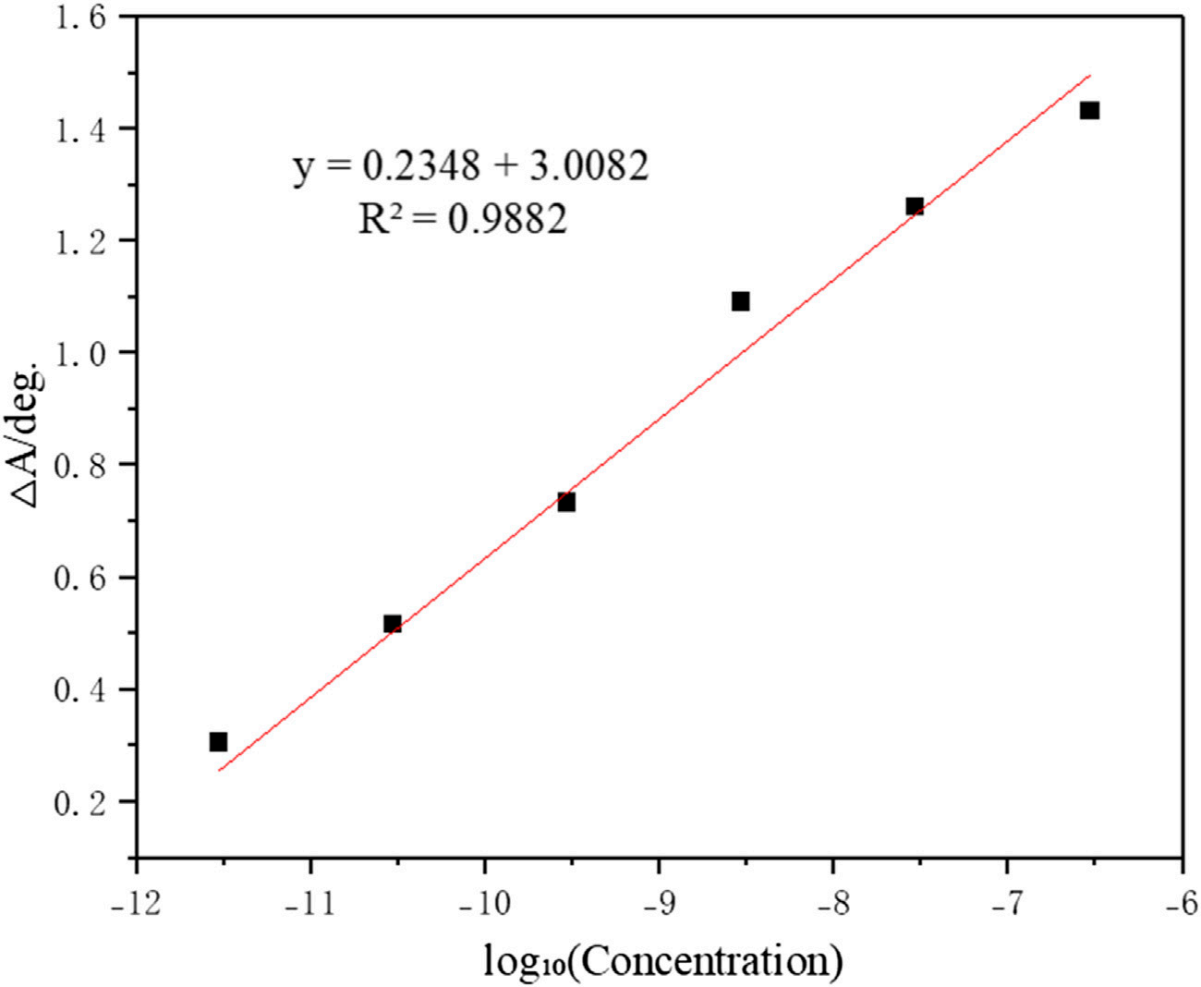
Calibration curve showing linear correlation between the resonance angle shift (ΔA) and logarithmic concentration after analyte adsorption.

Furthermore, we investigated the relationship between the resonance angle shift and adsorbed concentration following exposure to solutions of varying concentrations. The correspondence between the resonance angle shift and concentration is depicted in Figure 8. As shown in Figure 8, a linear relationship exists between the resonance angle shift and the logarithm of the concentration of methamphetamine hydrochloride aqueous solution. The linear equation is y = 0.2348x + 3.0082 (R^2^ = 0.9882). This may be due to the low solute content in the aqueous solution of methamphetamine hydrochloride used for testing. Within the tested concentration range, methamphetamine hydrochloride primarily exists in the form of hydrated ions in the aqueous solution. The hydrated methamphetamine cations significantly affect the formation of hydrogen bonds with the molecularly imprinted membrane by occupying donor sites, creating steric hindrance, and mediating indirect interactions. As a result, within the tested range, the change in the resonance angle is not linearly correlated with the solution concentration. The logarithmic transformation of the solution concentration occurs to mitigate the nonlinear effect of the concentration span on the resonance angle shift (Liu G. et al., 2018; Zhang et al., 2021).

**FIGURE 9:**
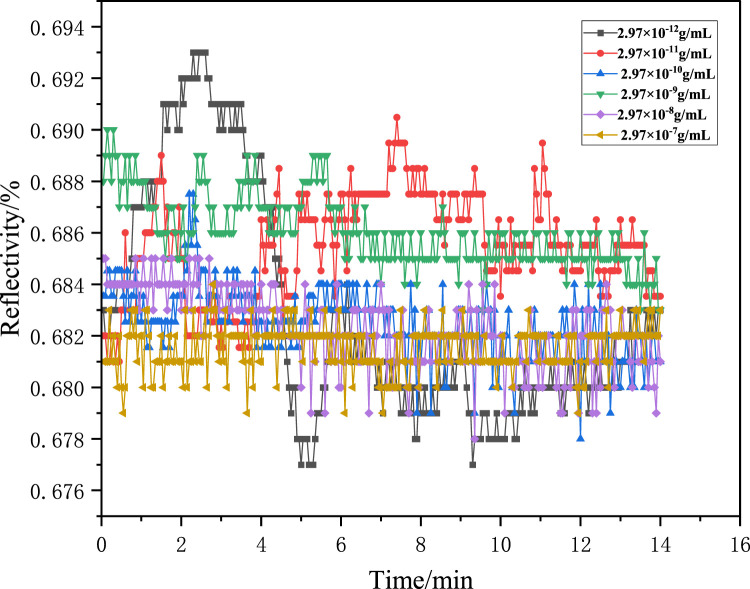
Adsorption kinetics curve of the NIP sensor chip.

Similarly, the NIP film was used to adsorb the solution with the abovementioned concentrations, and the reflectivity of NIPs changed irregularly, as shown in Figure 9. The NIP film displayed disordered adsorption behavior for methamphetamine hydrochloride (Figure 9), contrasting sharply with the ordered binding isotherm of MIPs. This divergence originates from nonspecific interactions due to recognition-site deficiency in NIPs.

### 4.4 Reusability

**FIGURE 10:**
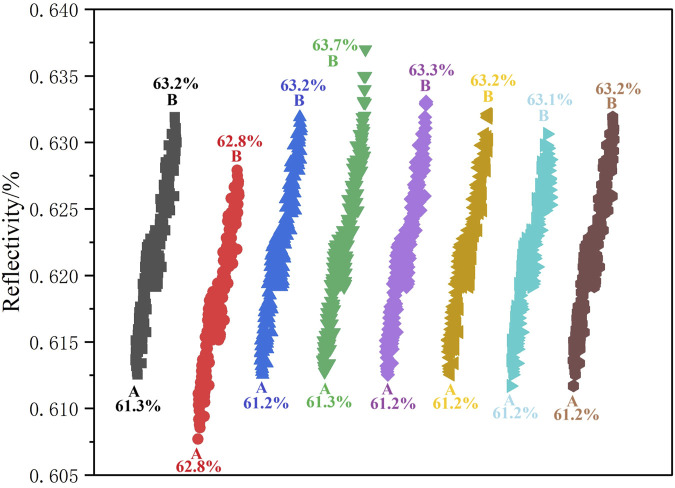
Adsorption–elution repeatability test curve of the SPR sensor chip.

Repeatability of the SPR sensor chip was tested using the acetic acid eluent and 2.97 × 10^−11^ g/ml methamphetamine hydrochloride solution. The initial reflectivity is adjusted to approximately 60%, with each adsorption step lasting approximately 10 min. The change in maximum reflectivity during the adsorption–elution cycle was observed, as shown in Figure 10. The results showed that the maximum reflectivity of the SPR sensor chip was not significantly reduced after eight cycles of adsorption. Each curve in Figure 10 represents the change in reflectivity values from left to right for the first to eighth adsorption tests, respectively. It demonstrated that the single-layer methamphetamine hydrochloride MIF had excellent reusability. (Note: A and B in the figure represent the initial reflectivity and final reflectivity for each adsorption cycle, respectively.).

### 4.5 Stability

Five sensor chips were randomly selected to study the stability of the sensor chips, and the results are shown in Table 2. When different chips were used to adsorb a 2.97 × 10^−11^ g/mL methamphetamine hydrochloride solution, the relative standard deviation (RSD) of the reflectivity-changed value was 3.7%, showing that the synthesized sensor chip had good repeatability.

**TABLE 2:** Comparative experimental data on the stability of the SPR sensor chip.

Chip number	Adsorption solution	Flow rate	Reflectivity changed value
1	2.97 × 10^–11^ g/ml methamphetamine hydrochloride solution	10 rpm	2.13%
2			2.11%
3			2.13%
4			1.99%
5			1.98%
Relative standard deviation			3.70%

**FIGURE 11:**
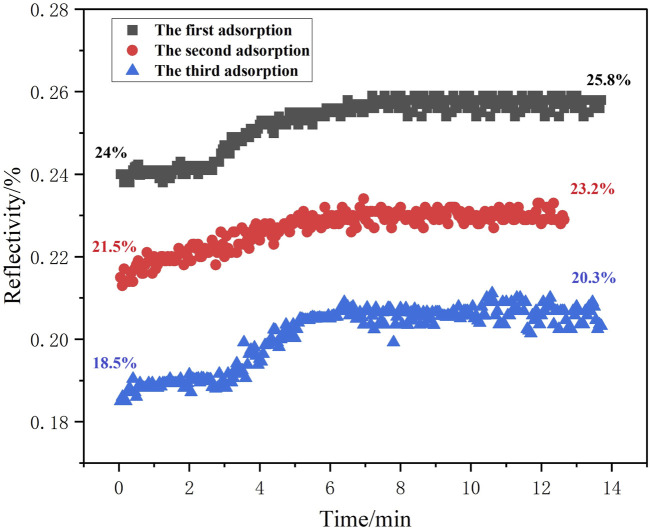
Replication stability analysis of the sensing chip.

Three replicate adsorption measurements were performed on a single chip with 2.97 × 10^−12^ g/mL methamphetamine hydrochloride solution. Following each binding event, the chip surface was regenerated and re-equilibrated to baseline conditions prior to subsequent injections. The operational stability of the chip was evaluated by comparing the degree of similarity in reflectivity changes across three consecutive adsorption experiments as Figure 11. Chip stability was assessed through triplicate adsorption measurements at differential initial light intensities (18%, 21%, and 24%) under isothermal conditions (25 °C ± 0.5 °C), with outcomes visualized in Figure 11. The reflectivity of the chip maintained a consistent 1.8% increase during testing. The identical adsorption curve profiles validated the robust operational stability of chips fabricated using this synthesis method.

**FIGURE 12:**
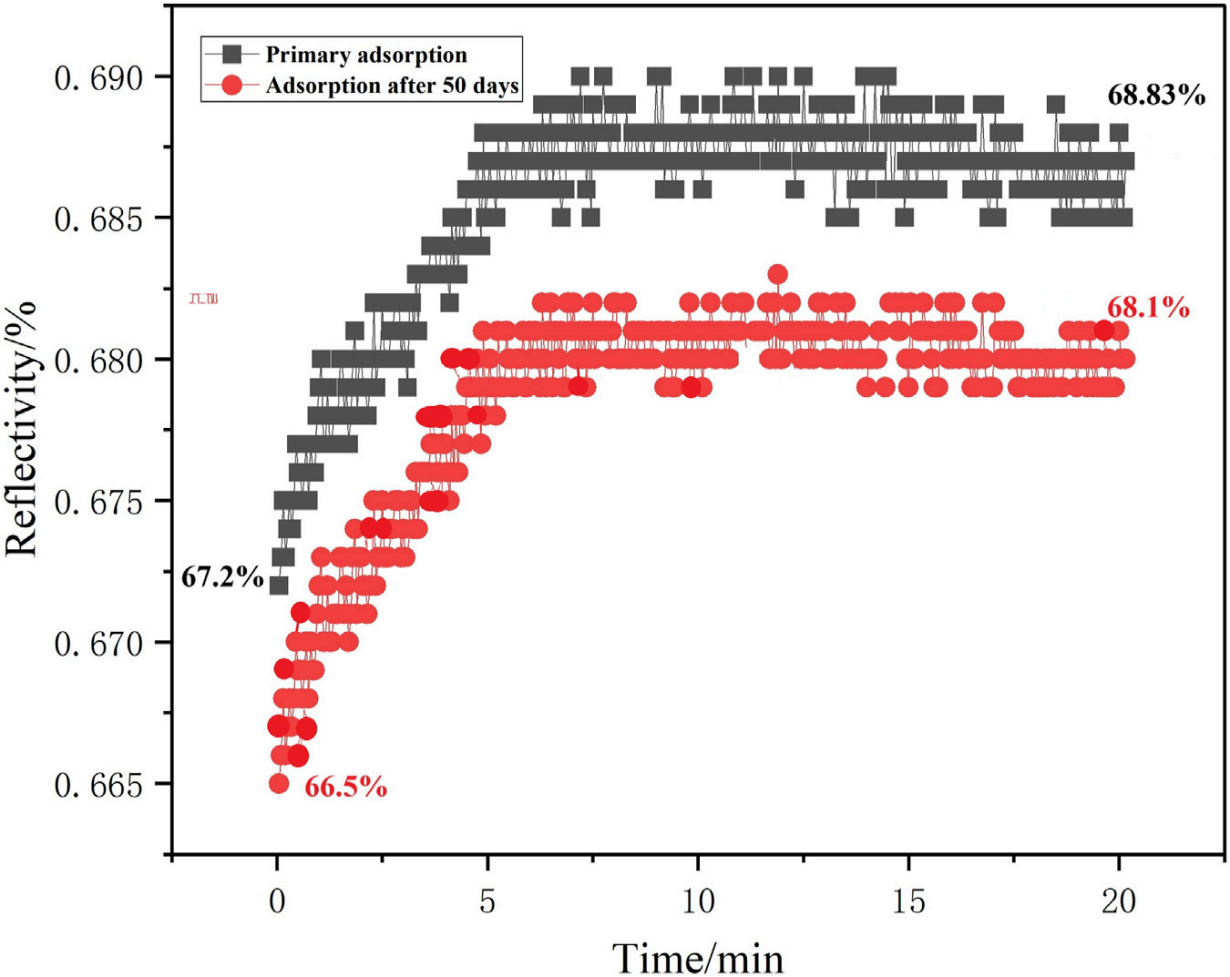
The stability adsorption experiment of the SPR sensor chip.

The sensor chip placed for a long time was tested to detect its stability. As shown in Figure 12, chips aged for 50 days were exposed to a 2.97 × 10^−12^ g/mL (0.003 ng/mL) methamphetamine hydrochloride solution to determine whether their detection sensitivity had undergone significant changes. The reflectivity changed value was 98.5% in the initial experiment, which proved that the synthesized sensor chip had outstanding stability.

### 4.6 Target-selective performance evaluation for SPR chips

**FIGURE 13:**
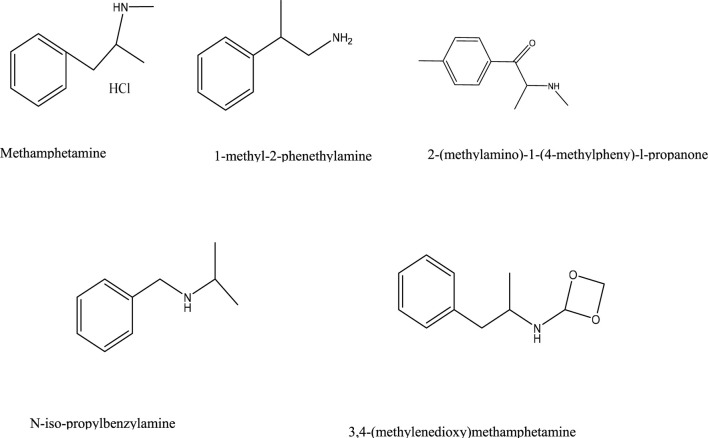
Chemical configurations of selected structural analogs.

The selective specificity of molecularly imprinted films serves as a critical parameter for evaluating the chip performance. To assess this property in the synthesized films, four structural analogs of methamphetamine hydrochloride were selected for adsorption experiments: 1-methyl-2-phenethylamine, 2-(methylamino)-1-(4-methylpheny)-l-propanone, 3,4-(methylenedioxy)methamphetamine, and N-iso-propylbenzylamine. Identical experimental protocols and conditions to those used for methamphetamine hydrochloride adsorption were maintained, with only the analyte species varying. Structural formulas of these compounds are provided in Figure 13.

**FIGURE 14:**
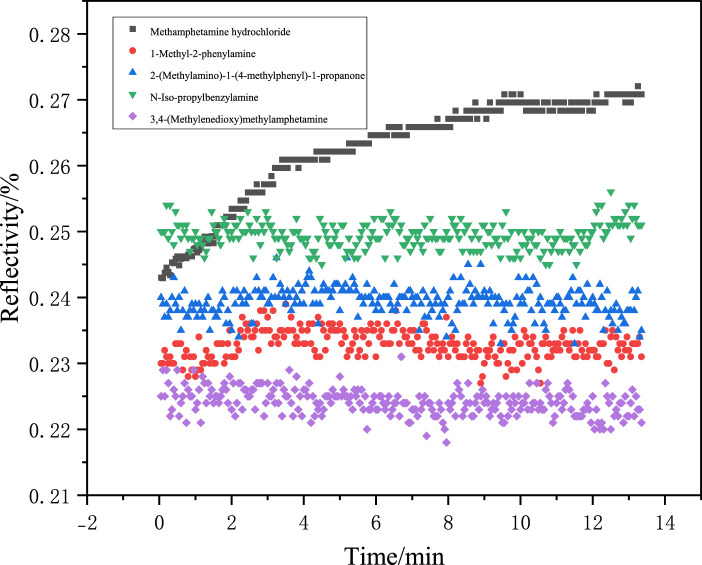
Adsorption curves of chemical structural analogs.

Aqueous solutions of the four analogs were prepared at identical concentrations (2.97 × 10^−8^ g/mL). As shown in Figure 14, the synthesized molecularly imprinted chips exhibited minimal adsorption toward structural analogs, with reflectivity increases below 0.5% throughout the testing period. In contrast, a significant 2.5% reflectivity enhancement was observed for methamphetamine hydrochloride solution under identical conditions. These results demonstrate excellent chip specificity toward the target analyte.

### 4.7 Detection of methamphetamine hydrochloride in dust

The methamphetamine hydrochloride in dust was dissolved with water to prepare 1.6 × 10^−10^ g/mL standard solution and then diluted with distilled water to 1.6 × 10^−11^ g/mL and 1.6 × 10^−12^ g/mL solutions. The test results are shown in Table 3. The sample recovery rates were 101.7%, 97.3%, and 99.6%, respectively. In short, the SPR sensor chip synthesized in this paper can be used for qualitative detection of methamphetamine in dust.

**TABLE 3:** Detection of methamphetamine in dust using an SPR sensor chip.

No.	Theoretical concentration (g/ml)	Initial detection value (g/ml)	Addition amount (g/ml)	Detection data after adding (g/ml)	Recovery rate/%
1	1.6 × 10^−10^	2.17 × 10^−10^	3.98 × 10^−10^	6.22 × 10^−10^	101.7
2	1.6 × 10^−11^	2.32 × 10^−11^	4.53 × 10^−11^	6.73 × 10^−11^	97.3
3	1.6 × 10^−12^	2.04 × 10^−12^	4.12 × 10^−12^	6.14 × 10^−12^	99.6

### 4.8 Advantages

Compared with the sensor chip of the similar drug detection product in the market, the SPR drug detection chips have the following advantages. For example, the sampling range is wide, and it can detect various forms of substances such as gas, dust, and liquid. In addition, the detection limit of methamphetamine hydrochloride can reach 2.97 × 10^−12^ g/mL. In addition, the detection results can be displayed quickly, and the duration of a single detection is approximately 6–10 min. Finally, online detection can be realized, and the detection results can be uploaded to the anti-drug network in real time, providing the police with timely alerts. Performance comparisons of sample adaptability, sensitivity (LOD), and operational speed between the developed approach and reference methods are provided in Table 4.

**TABLE 4:** Comparative analysis of detection method parameters.

Analytical method	Environmental sample	Detection limit	Response time	Ref
GC	Urine	200 μg/ml	15–30 min	Zhai et al. (2014)
GC-MS/MS	Hair	1.5 ng/mg	10–30 min	Ni et al. (2020)
HPLC	Solid Powder	0.52 μg/L	15 min	Fu et al. (2007)
Colloidal gold immunochromatography	Saliva	50 ng/ml	3–8 min	Chen et al. (2022)
Enhanced Raman spectroscopy	Drinks	100 μg/ml	3–5 min	Zhang et al. (2011)
Bioanalysis	Urine	1000 μg/L	3–5 min	Jiang et al. (2004)

## 5 Equipment testing

### 5.1 Equipment system

We develop a variety of drug rapid detection equipment to detect various forms of substances based on the MIT and SPR technology. The equipment can be used in multiple application scenarios. Moreover, the equipment enables precision and trace detection, along with real-time remote transmission of data.

**FIGURE 15:**
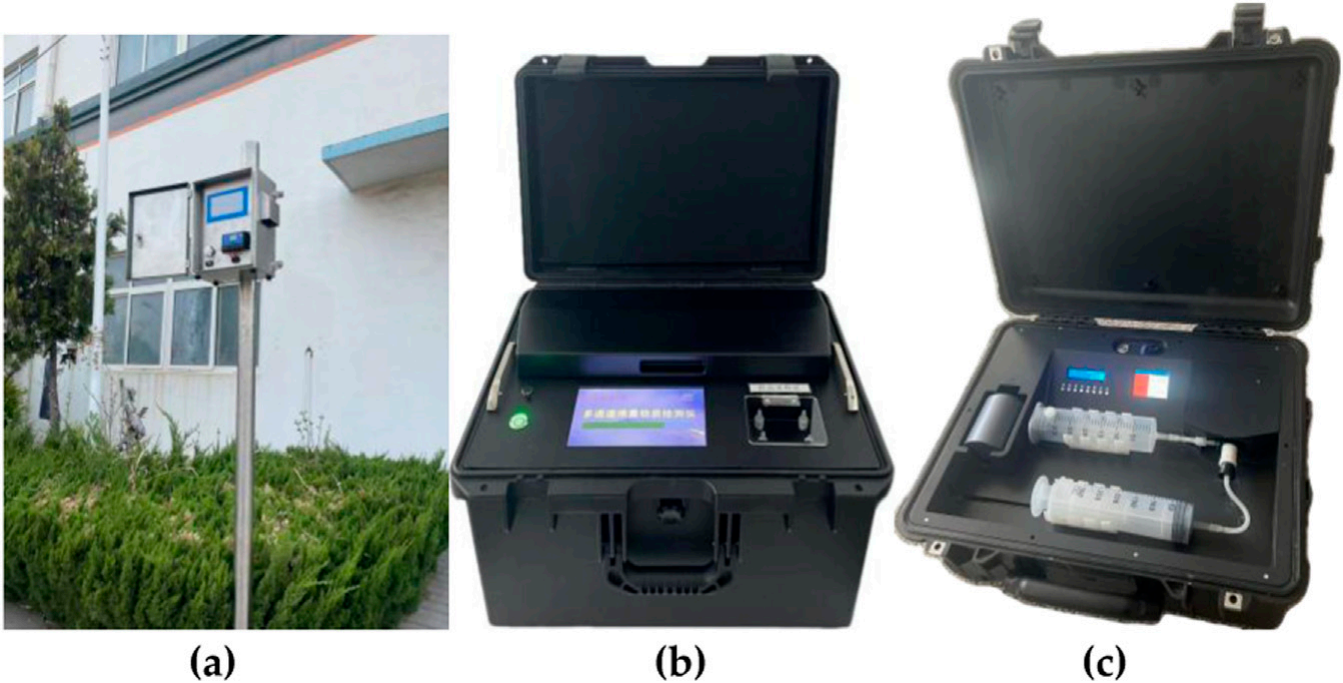
These are three devices based on MIT and SPR technologies. **(a)** Online controlled substance detector for wastewater; **(b)** multi-channel-prohibited substance sampling detector, and **(c)** portable drug detector.

Equipment 1: “Online controlled substance detector for wastewater.” This equipment is fixed in a certain place to be used to locate drug-related sites. The core of the equipment is fixedly installed beside the sewage outlet of the community or entertainment place, and the sampling head is immersed in the sewage, as shown in Figure 15a.

Equipment 2: “Multi-channel prohibited substance sampling detector.” There are two types of devices: automatic and semi-automatic for rapid detection of sewage water samples. Water samples can be collected from the water outlet of the unit to identify the drug-related unit in the community where drug use may be occurring. The equipment is shown in Figure 15b.

**FIGURE 16:**
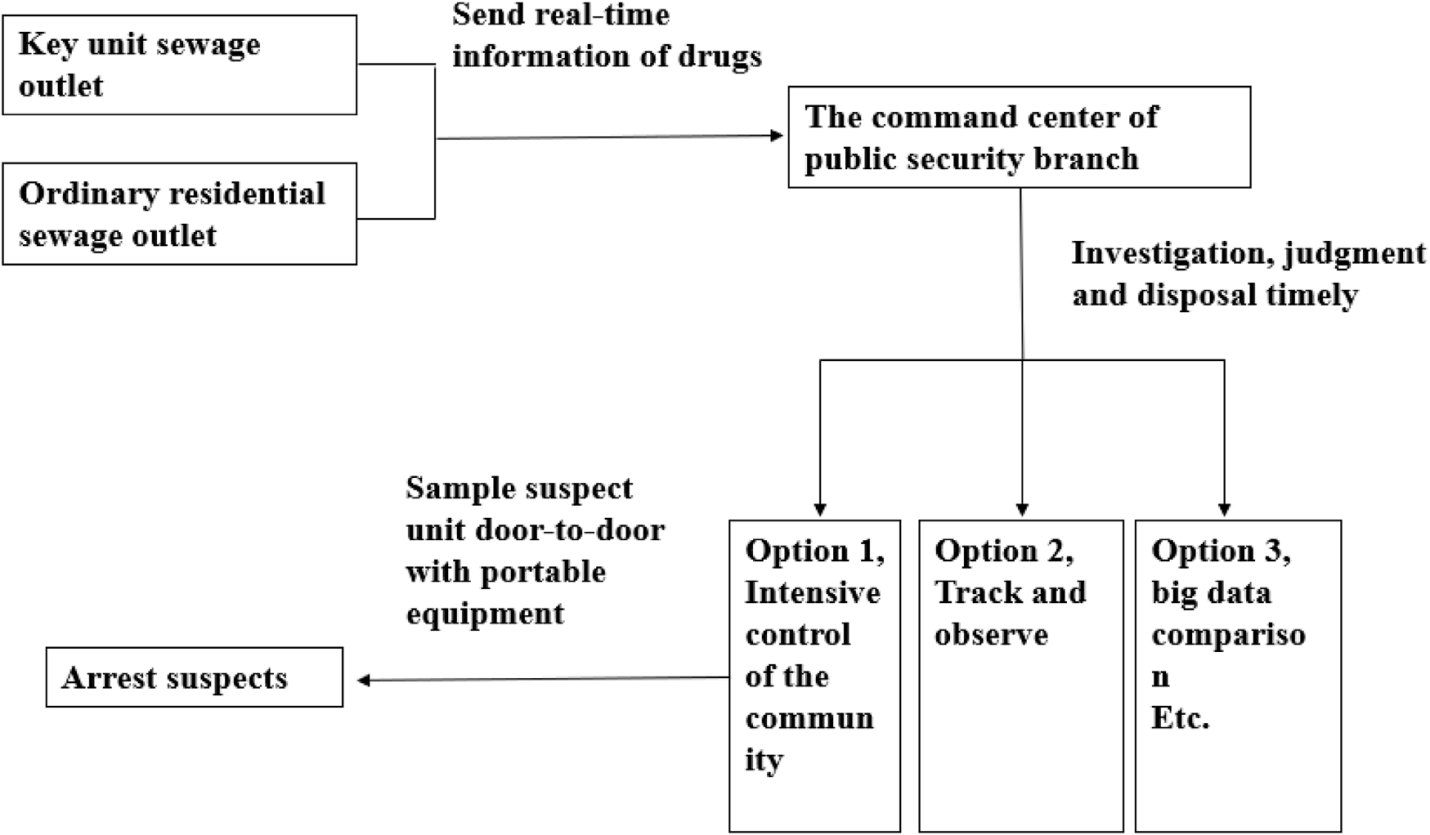
Schematic diagram of digital intelligent detection and control.

Equipment 3: “Portable drug detector.” This equipment is mainly used to detect the drug residue powder. When the drug-related unit is locked on, the equipment is used to take samples of some substances on the doorknob. It aims to identify the drug-related persons and take appropriate measures. The equipment is shown in Figure 15c. The schematic diagram of digital intelligent drug prevention and control is displayed in Figure 16.

All the above equipment can transmit data to the online platform of drug monitoring by wireless networking. First, the residence with drug addicts is locked using the equipment of the drug online monitor. Second, the unit is locked using the equipment for rapid inspection of the drug in sewage. Finally, the tenements are locked using the portable drug detector. The whole process, relying on the system to find clues and assist decision-making, can narrow the scope and lock the suspect. At the same time, the detector can make detections in real time for 24 h. When someone is taking drugs or producing drugs in the community, the detection signal will be transmitted to the server that is managed by police. Then, the command center will take appropriate measures based on the information.

### 5.2 Equipment detection capability

The aforementioned equipment is used for real-time online detection of environmental sewage, sewage sample testing, and detection of dust-like suspicious substances. Each of them presents itself characteristics. The multi-channel prohibited substance sampling detector was used for introduction, as shown in Figure 15b.

The multi-channel prohibited substance sampling detector is very simple to operate. No pretreatment is required for samples. The test results will be displayed within 6 min after the sample is directly dropped into the sample pool and the detection button is pressed. The detection accuracy is as high as 10^-13^ mol/L. The application fields of the equipment include screening drug addicts in entertainment places, drug driving, drug trafficking inspection, regular review of drug addicts, drug abuse inspection of airline pilots, drug inspection of logistics companies, and drug parcel inspection at customs. The technical specifications of the multi-channel prohibited substance sampling detector are shown in Table 5.

**TABLE 5:** Technical specifications of the multi-channel prohibited substance sampling detector.

Technology	MIT and SPR
Detectable substance	Methamphetamine, morphine, and heroin (Add as needed)
Sampling method	Sample liquids such as saliva, urine, domestic sewage, and natural water samples
Sensitivity	10^−13 ^mol/L
Alarm method	Sound and light alarm
Analysis time	6–10 mins
False alarm rate	<1% (keep the temperature constant)

### 5.3 Equipment detection capability

#### 5.3.1 Technical principle

The core component of the online controlled substance detector for wastewater is the sensing chip. The MIF loaded with the molecular pores of methamphetamine hydrochloride exhibits significant SPR after binding to the target molecule, resulting in a decrease in the reflected light intensity. When the reflected light intensity decreases to the preset value, the device issues an alarm signal and remotely transmits the data information to the police command center, providing technical reference for anti-drug work.

#### 5.3.2 Sample processing and collection

The X Residential Community was selected for the drug detection test. In the toilet of House A in this community, 300 mL, 150 ng/mL, 300 ng/mL, and 450 ng/mL solutions of methamphetamine hydrochloride were respectively poured. The detection was carried out at intervals of 7 days for each concentration. The online controlled substance detector for wastewater was installed at the main sewage outlet of this community and conducted automatic sampling and detection every 60 min and continuously detected for 24 h.

#### 5.3.3 Sample detection

The online controlled substance detector for wastewater automatically transports the samples to the detection pool. The fast detection equipment displays the detection results based on the changes in the SPR light intensity caused by the specific recognition of the target molecule and transmits the results remotely to the police command center. If the device identifies the target molecule, it will automatically trigger an alarm and save a wastewater sample for subsequent testing by law enforcement personnel.

#### 5.3.4 Detection results

**FIGURE 17:**
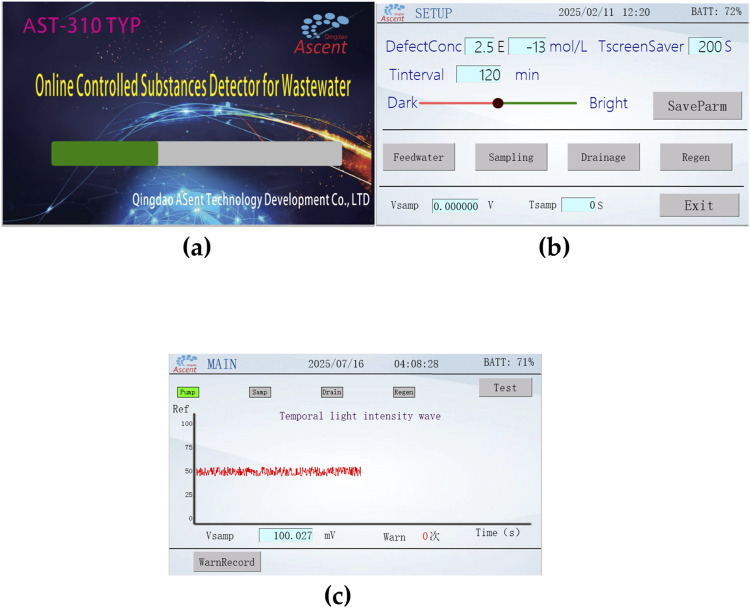
several interfaces of the online controlled substance detector for wastewater. **(a)** Startup interface; **(b)** setting interface; **(c)** detection interface.

As shown in Table 6, for 150 ng/mL, 300 ng/mL, and 450 ng/mL concentrations of methamphetamine hydrochloride placed in the bathroom of the resident, the online controlled substance detector for wastewater was able to raise alarms successively after multiple rinses, which is in line with the product expectation. Figures 17a–c present the startup, setting, and detection interfaces of the online controlled substance detector for wastewater. In addition, practical tests of this device are underway.

**TABLE 6:** Results of the online controlled substance detector for wastewater.

Time	Concentration ng/mL		
h			
	150	300	450
1	Normal	Normal	Normal
2	Normal	Normal	Normal
3	Normal	Alarm	Alarm
4	Normal	Alarm	Alarm
5	Alarm	Alarm	Alarm
6	Alarm	Alarm	Alarm
7	Alarm	Alarm	Alarm
8	Alarm	Alarm	Alarm
9	Alarm	Alarm	Alarm
10	Alarm	Alarm	Alarm
11	Alarm	Alarm	Alarm
12	Alarm	Alarm	Alarm
13	Alarm	Alarm	Alarm
14	Alarm	Alarm	Alarm
15	Alarm	Alarm	Alarm
16	Alarm	Alarm	Alarm
17	Alarm	Alarm	Alarm
18	Alarm	Alarm	Alarm
19	Normal	Alarm	Alarm
20	Normal	Alarm	Alarm
21	Normal	Alarm	Alarm
22	Normal	Normal	Alarm
23	Normal	Normal	Alarm
24	Normal	Normal	Normal

## 6 Conclusion

The sensor chip and rapid drug detection equipment based on molecular imprinting and SPR technology have the characteristics of high precision, multi-state detection, low professional dependence, and strong anti-interference. This series of equipment can play a huge role in the rapid detection of drugs. The main technical features are as follows:

1. High-precision detection can be realized. This series of equipment combining the unique advantages of SPR can improve the detection accuracy to 10^-12^ g/mL.
2. It can realize direct detection in the natural environment. The equipment for detecting prohibited substances in sewage has been put into practical use in anti-drug brigades in cities like Chongqing and Wuhan. The public security authorities in these two places have highly praised the experimental results of this equipment.
3. No large expensive professional equipment or professionals are required in this study. In the traditional technology, the expensive equipment is operated by professionals, and the consumables are more than 10 kinds of chemical reagents. In addition, the equipment needs to be calibrated and maintained regularly. The new technology is completely localized, the equipment is portable, and the service cycle is maintenance-free. It meets the demands of one-button operation, on-the-spot, real-time, and high-precision detection.
4. Anti-interference reduces the false alarm. The new technology has high selectivity and strong ability to identify and is basically unaffected by acid, alkali, heat, and other factors. It can minimize and even eliminate false alarms, which is of great significance to reduce the labor intensity of police officers and reduce the disputes caused by misjudgment. In the post-field-test evaluation report issued by the Chongqing Municipal Police Department, the online wastewater narcotics detector received high appraisal for its testing accuracy rate.
5. The consumables feature low costs. Depending on the target analyte, sensing chip prices range from tens to thousands of RMB, with each chip being regenerable approximately 10 times. This translates to a per-test cost spanning several to hundreds of RMB.
6. Such devices have the following limitations: (1) single-analyte detection per chip—inability to simultaneously detect multiple substances on one chip; (2) mandatory 20-min re-equilibration after chip replacement before stable monitoring is resumed; (3) continuous monitoring interruption during chip replacement and system re-equilibration. We are actively addressing current limitations through iterative hardware optimization and algorithmic enhancements to enable multiplexed detection and minimize system downtime.

## Data Availability

The original contributions presented in the study are included in the article/Supplementary Material; further inquiries can be directed to the corresponding author.
