# Supplementary material for: Rapid drug detection equipment based on molecular imprinting and surface plasmon resonance technology
Source: Front Chem. 2025 Sep 11;13:1645843. doi: 10.3389/fchem.2025.1645843 (PMC12462404; doi:10.3389/fchem.2025.1645843)
Supplement: Supplementary file 1 [file DataSheet1.pdf]

The ratio of functional monomer to template molecule is the core factor determining the performance of molecularly imprinted polymers, directly affecting the quantity, quality, and selectivity of imprinting sites. The functional monomer is the key component for forming specific binding cavities.

Insufficient quantity of functional monomers hinders sufficient interaction with template molecules, leading to a dramatic reduction in the number of effective imprinting sites. Consequently, the adsorption capacity of molecular imprinting polymers may be significantly reduced, indicating decreased loading capacity of target molecules per unit polymer. Furthermore, insufficient functional monomer relatively increases the proportion of cross-linker, resulting in an overly dense polymer network. This causes difficulties in template molecule elution, increases template residue, amplifies mass transfer resistance for specific recognition, hinders the diffusion of target molecules to binding sites, and ultimately reduces recognition efficiency.

When functional monomer is in excess, some monomers fail to bind with template molecules and randomly form non-imprinted cavities during polymerization. The adsorption capacity may appear to increase, but in reality, this is a false impression created by low-affinity binding sites. Secondly, acrylic functional monomers (like methacrylic acid) contain flexible groups such as methylene and carboxyl groups, which enhance the deformation ability of the imprinted cavities, preventing them from maintaining conformations precisely complementary to the template molecule. Additionally, the dense network or disordered cavities formed by excess monomer impede template molecule diffusion, significantly reducing adsorption/desorption rates and making it difficult to reach binding equilibrium.

Hydrogen Bonding

In acetonitrile, methamphetamine hydrochloride and methacrylic acid can theoretically form a maximum of 4 hydrogen bonds (3 from $-NH_3^+$ with carboxyl oxygens, and 1 from -COOH with Cl^-). The actual number may be slightly reduced due to steric hindrance, but the maximum possible is 4. In this study, an experimental screening approach was employed: a series of sensing chips with different template molecule to functional monomer ratios were synthesized. The optimal ratio was determined by testing the adsorption sensitivity of these chips.

Experimental Approach

Chips were synthesized with template molecules: functional monomer (molar ratio) = 1:3, 1:4, 1:5, 1:6, and 1:8. Based on previous research findings [1], the amount of cross-linker used was 10 times the molar amount of the template molecule. This cross-linker ratio ensures optimal mechanical strength and recognition specificity for the molecular imprinting polymers. These synthesized chips were then used to adsorb a methamphetamine hydrochloride solution

with a concentration of 2.97×10^{-12} g/ml. The adsorption sensitivity of the different chips was compared. The figure below shows the adsorption curves for the different template molecule to functional monomer ratios.

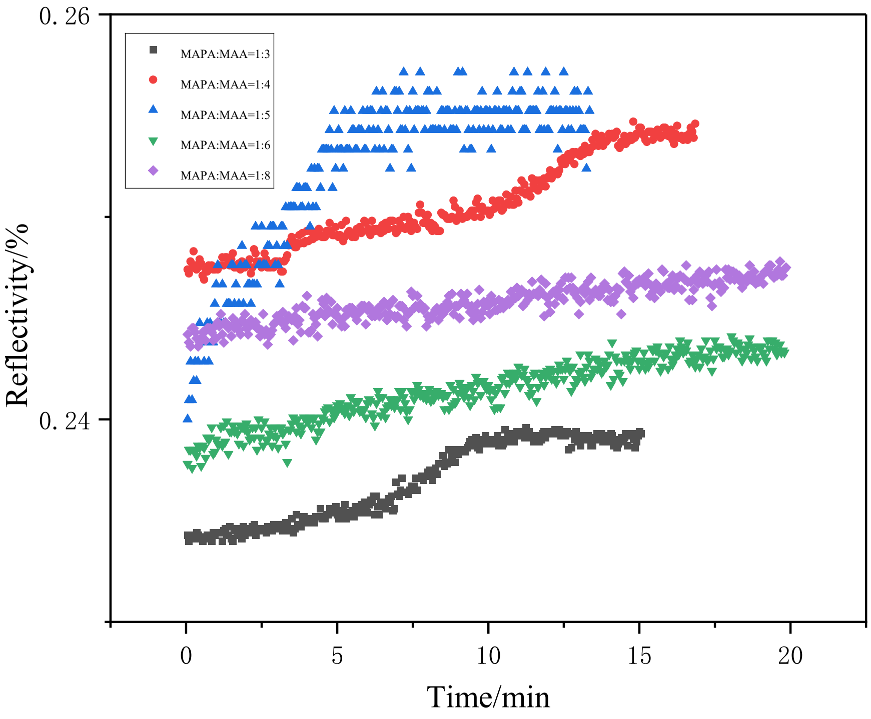

Figure 1. Adsorption curves for various molar ratios of template molecule to functional monomer.

Table 1. Comparative data of adsorption curves for different molar ratios of template molecule to functional monomer.

Template molecule: Functional monomer	Adsorption equilibrium time /min	Reflectivity change /%
1:3	10	0.5
1:4	14	0.7
1:5	5	1.6
1:6	20	0.6
1:8	> 20	0.35

Note: This refers to the molar ratio of template molecules to functional monomers.

Based on Table 1 and Figure 1, when the template molecule : functional monomer ratio is below 1 : 5, the reflectivity change of the chip increases with higher molar amounts of functional monomer upon adsorption of identical solution concentrations, resulting in enhanced chip sensitivity. This may be because as the number of functional monomers increases, the "fixed"

template molecules also increase, leading to more available binding sites, thus making the chip more sensitive. When the ratio of template molecules to functional monomers is 1:5, the optimal proportion is achieved where the adsorption equilibrium time is the shortest and the reflectance variation reaches its maximum. When the template molecule to functional monomer ratio exceeds 1:5, an excess of functional monomers may lead to the formation of a dense molecular network in the molecularly imprinted film. This can hinder template molecule diffusion and ultimately prolong the adsorption equilibrium time.

References

1. Yuan Tan, Study on surface plasmon resonance sensor based on double-layer molecular imprinting polymer film [D]. Beijing Institute of Technology, China, 2015.
